# In vitro fermentation potential of undigested dietary protein

**DOI:** 10.1093/jas/skaf119

**Published:** 2025-04-17

**Authors:** Hanlu Zhang, John W Cone, Arie K Kies, Wouter H Hendriks, Nikkie van der Wielen

**Affiliations:** Animal Nutrition Group, Department of Animal Sciences, Wageningen University & Research, Wageningen, The Netherlands; State Key Laboratory of Animal Nutrition, College of Animal Science and Technology, China Agricultural University, Beijing, China; Animal Nutrition Group, Department of Animal Sciences, Wageningen University & Research, Wageningen, The Netherlands; ArieKiesAdvies, Druten, The Netherlands; Animal Nutrition Group, Department of Animal Sciences, Wageningen University & Research, Wageningen, The Netherlands; Animal Nutrition Group, Department of Animal Sciences, Wageningen University & Research, Wageningen, The Netherlands; Division of Human Nutrition and Health, Department of Agrotechnology and Food Sciences, Wageningen University & Research, Wageningen, The Netherlands

**Keywords:** curve fitting, gas production system, pig, plant protein, protein fermentation

## Abstract

This study aimed to investigate the in vitro fermentation potential of ileal digesta from pigs fed 7 protein sources with different batches—maize germ meal (**MGM**), cottonseed meal (**CSM**), rapeseed cake (**RSC**), rapeseed meal (**RSM**), peanut meal, soybean meal (**SBM**), and sunflower meal (**SFM**)—to assess their potential impact on hindgut protein fermentation, which can be harmful to animals. Ileal digesta samples were incubated with porcine fecal inoculum under N-free conditions, with whey protein isolate (**WPI**) as a control. Gas production (**GP**) resulting from protein fermentation was monitored over 48 h and analyzed using a modified biphasic model to assess substrate fermentation and microbiota turnover dynamics. Significant variations in fermentation characteristics, including maximum GP rates (*R*_max_), cumulative GP, and microbiota turnover slopes, were observed across the protein sources. *R*_max_ values ranged from 16.8 ± 0.6 to 27.9 ± 0.8 mL/h for MGM and SFM, respectively compared to 12.5 ± 0.4 mL/h for WPI. Solubility and molecular mass analyses showed differences in the proportion of insoluble nitrogenous molecules and the distribution of soluble molecules, reflecting varying fermentability. Standardized hindgut fermentation potential differed significantly among the protein sources, with MGM exhibiting the highest potential (1.18 L/g dietary protein) and SBM the lowest (0.46 L/g dietary protein). These findings provide valuable insights into the differential accessibility of undigested protein to hindgut microbiota, contributing to more effective diet management and optimization of animal and human nutrition strategies.

## Teaser

The indigestible protein of 7 dietary protein sources for pigs differ in their fermentation potential, offering valuable insights into optimizing nutrition strategies.

## Introduction

Undigested dietary and unabsorbed endogenous proteins, peptides, and amino acids (**AA**) entering the large intestine can become substrates for degradation and utilization by the gut microbiota (Smith and Macfarlane, 1998). This fermentation process of proteinaceous material can result in the production of detrimental metabolites, such as ammonia, various amines, phenolic compounds, sulfides, and branched-chain fatty acids (Gilbert et al., 2018). Understanding protein fermentation dynamics in the hindgut is important for addressing health risks from harmful protein-derived metabolites especially when dietary protein sources are less digestible or endogenous protein losses at the end of the small intestine are increased. Insights from animal models, such as pigs, can offer valuable parallels for understanding similar processes in humans, notably microbial metabolism and its health implications.

Efficient utilization of dietary protein sources is of critical importance in modern pig production due to its profound impact on growth performance, animal health, economic viability, and environmental impact of the industry (Nijdam, Rood, and Westhoek, 2012). Nutrient composition, variability, digestibility, and antinutritional factors are essential characteristics of protein sources for inclusion in such diets. In pigs, most of the ingested dietary protein will be digested and absorbed in the small intestine (Davila et al., 2013). Therefore, the standardized ileal digestibility (**SID**) of crude protein (**CP**) and its individual AA is widely used in feed formulation. The SID values, ranging from 75% to 85% for common feed ingredients, reflect variations in composition and processing methods (Stein et al., 2001). There is a significant body of research which investigated how dietary protein sources impact the intestinal microbiota composition, metabolites formed and, thereby, affect gut health in pigs (Htoo et al., 2007; Li et al., 2019; Schutkowski et al., 2019; Wu et al., 2022). Few studies, however, have investigated protein fermentation kinetics due to the difficulty of in vivo measurement. Predominantly, researchers have resorted to single-point in vivo measurement of protein fermentation-associated metabolites which is the net result of synthesis, breakdown, and absorption (Zhang et al. 2020). To study protein fermentation, an in vitro approach can provide more detailed information on the kinetics compared to in vivo studies where these processes are difficult to discern. Recently, we (Zhang et al. 2024) used a modified in vitro gas production (**GP**) technique (Cone et al., 2005) and a novel model for curve fitting to measure protein degradation kinetics, specifically focusing on the hydrolysis rate of the protein. The hydrolysis rate plays a crucial role in indicating N availability in the substrate for microbiota and demonstrates their fermentation potential since it is recognized as the rate-limiting step in fermentation (Vavilin et al., 2008).

Here, undigested and unabsorbed proteinaceous (dietary and endogenous) material present in ileal digesta from pigs fed 7 commonly used protein sources were assessed for their protein fermentation potential. The latter data on fermentation potential, in conjunction with the respective SID values of protein sources, were subsequently used to provide an estimate of the fermentation potential of individual dietary protein sources.

## Materials and Methods

Ileal digesta samples of individual pigs as well as detailed information on their composition were obtained from previous studies (Ma et al., 2019; Li et al., 2014; Li et al., 2015a, 2015b; 2017; Liu et al., 2015; Zhang et al., 2019) conducted in Beijing, China and transported in a freeze-dried form to Wageningen University (Wageningen, the Netherlands). For the additional in vitro analyses presented in this manuscript, no further ethical approval was required.

### Ileal digesta

The previous studies investigated the digestibility of different batches, cultivars or processed dietary protein sources. Samples originated from 7 separate studies investigating 10 batches of cottonseed meal (**CSM**), 8 batches of maize germ meal (**MGM**), 7 batches of peanut meal (**PM**), 4 batches of differently processed rapeseed cake (**RSC**), 9 batches of rapeseed meal (**RSM**), 11 batches of soybean meal (**SBM**) and 9 batches of sunflower meal (**SFM**). Each ingredient was grown at a different location in China during various years, except for the SBM, of which 5 batches originated from the US and Brazil. Each protein source was fed as the sole source of protein to ileal cannulated growing pigs in a 6 × 6 Latin square design with ileal digesta collected from 6 to 12 animals. All samples were freeze-dried and ground to pass a 0.25 mm screen and stored at −20 °C until transport and upon arrival at Wageningen University were stored at room temperature. Samples of the various pigs were pooled per ingredient, based on an equal quantity of the digestibility marker (Cr_2_O_3_).

### Chemical analyses

Analytical data were available for the original ileal digesta samples obtained from individual pigs before pooling including CP (GB/T 6432-2006), ether extract (GB/T 6433-2006), neutral detergent fiber (GB/T 20806-2006), acid detergent fiber (NY/T 1459-2007), ash (GB/T 6438-2007), calcium (Ca, GB/T 6436-2002), phosphorous (P, GB/T 6437-2002) and chromium (Cr, GB/T 13088-2006). For pooled samples, N level was analyzed using the Dumas method (ISO 16634) at Wageningen University for calculating sample weight in the fermentation studies. The AA composition of pooled samples was calculated based on the ratio of the amount pooled per digesta sample and the AA composition of individual samples obtained from previous corresponding studies. The amount of non-AA-N (NAAN) was calculated from total N level by subtracting the total AA-N (TAAN).

#### In vitro protein fermentation

For fermentation, a precisely weighed amount of pooled ileal digesta containing 10 mg N was incubated in 4 independent runs. Blank bottles without substrate as well as bottles containing whey protein isolate (**WPI**, Fonterra, New Zealand) were included in each run as controls. The in vitro protein fermentation procedure was performed as previously described (Zhang et al. 2024). Briefly, sealed bottles of 250 mL containing 60 mL of 2% pig fecal inoculum in a N-free buffer were prepared at the start of each run and incubated at 39 °C until the addition of the test substrate. The timing of the addition of substrate to the buffer-fecal mixture was determined by monitoring the GP of the blank bottles at 39 °C, which contained the same buffer-fecal mixture. This blank GP was recorded continuously using the method described in previous studies (Cone et al., 1996, 2005) until it reached a plateau after 1 to 2 h. Subsequently, the ileal digesta and control substrates were added to the different bottles and incubated in water baths at 39 °C for 48 h, with continuous recording of GP. The water level in the water baths was maintained throughout the fermentation period.

The buffer was supplemented with 21.56 g/L easily fermentable carbohydrates, namely 8.6 g maltose (M5885), 4.32 g pectin from citrus peel (P9135), 4.32 g xylose (X1500, all from Sigma-Aldrich, Saint Louis, USA) and 4.32 g soluble potato starch (Paselli WA4, Avebe food, Veendam, the Netherlands).

### Curve fitting

Ileal digesta samples showed a similar S-shape in GP curves, indicating a common pattern of this microbial process that fits with our assumption that after the pre-fermentation of buffer-inoculum mixture and the addition of the sample-containing bottle, the soluble N is accessible first to the microbiota. After all accessible N from the samples is utilized, GP is driven by the release of N through microbiota turnover. The GP of the blank group, thereby, only relates to microbiota turnover.

The GP data for each bottle, continuously recorded over 48 h, were used to calculate the: 1. lag time (*T*_lag_, h) of the start of fermentation (the time at which the cumulative GP of the substrate surpassed the cumulative GP of the blank within a run), 2. maximum GP rate (*R*_max_, mL/h) by dividing the gas released between 2 consecutive recorded time points by the time interval, 3. time when *R*_max_ occurred (*T*_*R*max_, h), 4. total GP generated from the protein source provided (GP_s_, mL/10 mg N) as determined by the model below, 5. time when GP_s_ occurred and microbiota turnover is assumed to start (T_GPs_, h) and 6. slope (mL/h) of the linear line fitted to the cumulative GP after T_GPs_. The model from our previous study(H. Zhang et al., n.d.) was used to fit the cumulative GP data of individual bottles:


$$\begin{array}{l} \left\{ \begin{array}{l} GP_{c}=\;\frac{A_{1}}{1+{\left(\frac{B_{1}}{T}\right)}^{C_{1}}}+\frac{A_{2}}{1+{\left(\frac{B_{2}}{T}\right)}^{C_{2}}}\;T\leq T_{GP_{s}}\;GP_{c}=\;\frac{A_{1}}{1+{\left(\frac{B_{1}}{T_{GP_{s}}}\right)}^{C_{1}}}+\frac{A_{2}}{1+{\left(\frac{B_{2}}{T_{GP_{s}}}\right)}^{C_{2}}}+slope\times \left(T-T_{GP_{s}}\right)\;T>T_{GP_{s}}\; \end{array}\right. \end{array}$$

where, GP_c_ (mL/10 mg N) denotes the amount of gas produced per 10 mg N of sample incubated (also corrected by the GP of the blank groups at *T*_lag_) at time *T* after *T*_lag_, *A*_*i*_ (mL/10 mg N) represents the asymptotic GP, *B*_*i*_ (h) is the time after incubation at which half of the asymptotic amount of gas has been formed, and *C*_*i*_ is a constant determining the sharpness of the switching characteristic of the curve. The parameter *i* indicates the number of phases in the curve (*i* = 1, 2). The model was used to derive GP_s_, T_GPs_, and the slope.

To further compare the different sources, the apparent hindgut GP potential of a dietary protein source (AGP_pro_, L/g ingested CP) was calculated based on GP_s_ of their ileal digesta and the apparent ileal N digestibility (AID, %) obtained from the studies the ileal digesta samples originated from:


$$\begin{array}{l} AGP_{pro}\;=\;\left(1\;\frac{AID}{100}\right)\times \;\left(\frac{GP_{s}}{10\times 6.25}\right) \end{array}$$

The standardized GP potential of a dietary protein source (SGP_pro_, L/g ingested CP) was corrected for the GP potential of the corresponding endogenous proteins (GP_endo_, mL/10 mg N) obtained from our previous studies (Zhang et al. 2024) according to the following equation:


$$\begin{array}{l} SGP_{pro}=AGP_{pro}-\left(\frac{GP_{endo}}{10\times 6.25}\right)\times \left(\frac{SID-AID}{100}\right) \end{array}$$

For PM and RSM an average of GP_endo_ from the other 5 studies was used, as they were not available. Standardized values were further converted to percentages relatively to SBM (SGP_pro-SBM_, %). All values were averaged for different batches per protein source.

### Protein solubility and size exclusion chromatography

For each protein source, 3 different ileal digesta samples closest to the average *R*_max_ value of each protein source, were selected. For each pooled ileal digesta, a 1.5 g sample with a known N content was dissolved in 5 mL buffer as used for in vitro fermentation and vortexed before being centrifuged (30 min, 20,000 × *g*, 20 °C). The supernatant was analyzed for N content (Dumas) to calculate N solubility. Subsequently, 50 μL of the supernatant was used to determine the molecular mass distribution by size exclusion chromatography using an Akta pure 25 system with a Superdex 75 column. The eluent used was a 20 mM phosphate buffer with 150 mM NaCl, pH 7. The absorbance was measured at 214 and 280 nm and a calibration curve of the eluent volumes was established using blue-dextran (2,000 kDa), conalbumin (76 kDa), ovalbumin (42.7 kDa) and β-lactamase (28.9 kDa). The obtained chromatograms were separated into molecular weight ranges of 0 to 1, 1 to 2.5, 2.5 to 5, 5 to 10, 10 to 50 and >50 kDa based on the calibration curve at 214 nm.

### Statistical analyses

The values for *T*_lag_, *R*_max_, T_Rmax_, GP_s_, T_GPs_, and slope of ileal digesta samples and WPI were analyzed using a mixed model. In this model, protein source was considered a fixed factor, and replication run was treated as a random factor, except when there was a run effect. Differences between individual protein sources and the positive control (WPI) were assessed using Dunnett’s test. To compare differences between protein sources, Tukey’s test was employed. Ileal digesta samples collected from pigs fed diets containing various batches of the same protein source were considered nested factors when examining source effects. If the nested factor was found to be non-significant, it was removed, and the ingredient was then included as a random factor. Additionally, differences within protein sources were evaluated across different incubation runs for each batch, with replication run treated as random factor except when there was run effect.

To examine the relationship between gas fermentation parameters and molecular mass distribution of N in the ileal digesta samples, a stepwise general linear model selection was employed. All statistical analyses were conducted using SAS 9.4 (SAS Inst. Inc., Cary, NC, USA), with probability values < 0.05 considered significant and values < 0.1 considered a trend of significance. Group values were reported as means ± standard error.

## Results

### Chemical composition

The average N content of pooled ileal digesta samples from different batches (CSM, MGM, PM, RSC, RSM, SBM, SFM) was 24.2, 20.8, 32.3, 28.2, 28.2, 25.4, 19.0 mg/g DM, respectively. The calculated TAAN content of pooled samples is shown in Table 1 and was highest for CSM. The average nutrient composition and CP ileal digestibility of the different batches and the minimum and maximum values for a protein source are shown in Table S1. Detailed AA composition is provided in Table S2. The main differences between ileal digesta samples were observed for glutamic acid, methionine, lysine, and arginine.

**Table 1.** Mean (min, max) total nitrogen (N), total amino acid N (TAAN) and non-amino acid N (NAAN) of ileal digesta samples from pigs^1^ fed different dietary protein sources from different batches

Item Protein source	No of batches	N mg/g dry matter	TAAN mg/10 mg N	NAAN mg/10 mg N
Cottonseed meal	10	24.2 (20.0, 27.7)	10.2 (9.7, 11.5)	−0.16^2^ (−1.5, 0.35)
Maize germ meal	8	20.8 (19.0, 23.8)	7.1 (6.8, 7.5)	2.9 (2.5, 3.2)
Peanut meal	7	32.3 (22.7, 37.6)	8.4 (8.2, 8.8)	1.6 (1.2, 1.9)
Rapeseed cake	4	28.2 (25.6, 34.8)	8.2 (7.3, 9.1)	1.8 (1.0, 2.7)
Rapeseed meal	9	28.2 (26.4, 30.5)	8.2 (8.0, 8.4)	1.8 (1.6, 2.0)
Soybean meal	11	25.4 (23.3, 28.4)	8.8 (8.2, 9.6)	1.2 (0.4, 1.8)
Sunflower meal	9	19.0 (13.6, 22.9)	8.7 (7.9, 11.7)	1.3 (−1.7, 2.1)

^1^Each sample was pooled from 6 growing pigs fed the same protein source batch.

^2^Values < 0 are due to differences in N determination methods for pooled samples (Dumas) and the calculation of TAAN where AA composition from previous studies and pooling ratios are used. The molecular weight of each AA was adjusted, assuming 10% is in the free form (van der Wielen et al., 2023) and the remaining 90% with one water molecule deducted from the chain.

### Gas production of ileal digesta

**Figure 1.**
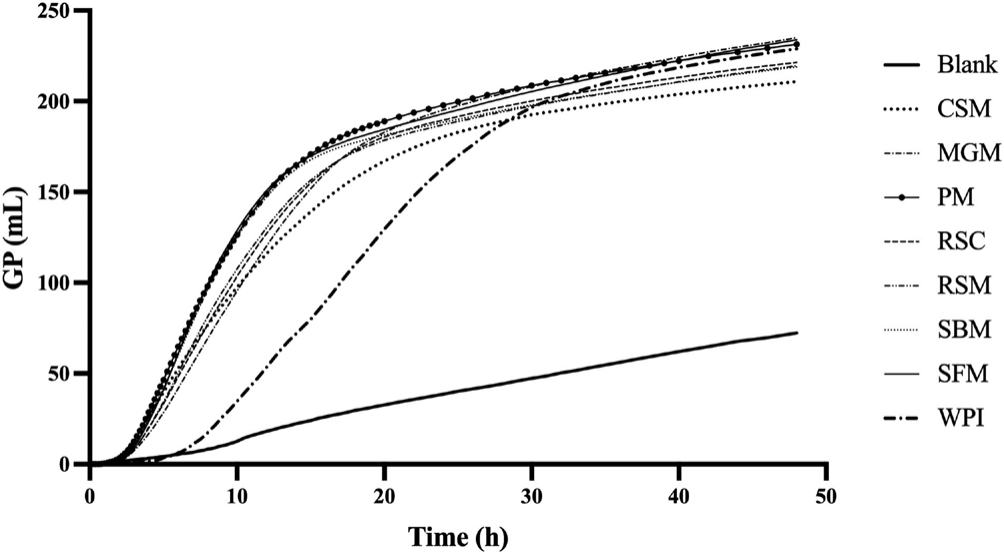
Measured 48 h in vitro cumulative GP of porcine fecal inoculum (Blank, *n* = 6), ileal digesta and WPI (*n* = 4). Ileal digesta were obtained from pigs in studies investigating the digestibility of different batches of cottonseed meal (CSM, *n* = 10), MGM (*n* = 8), rapeseed cake (RSC, *n* = 4), PM (*n* = 7), rapeseed meal (RSM, *n* = 9), soybean meal (SBM, *n* = 11) and sunflower meal (SFM, *n* = 9). All samples contained the same amount of nitrogen (10 mg) and were incubated in 4 runs.

**Figure 2.**
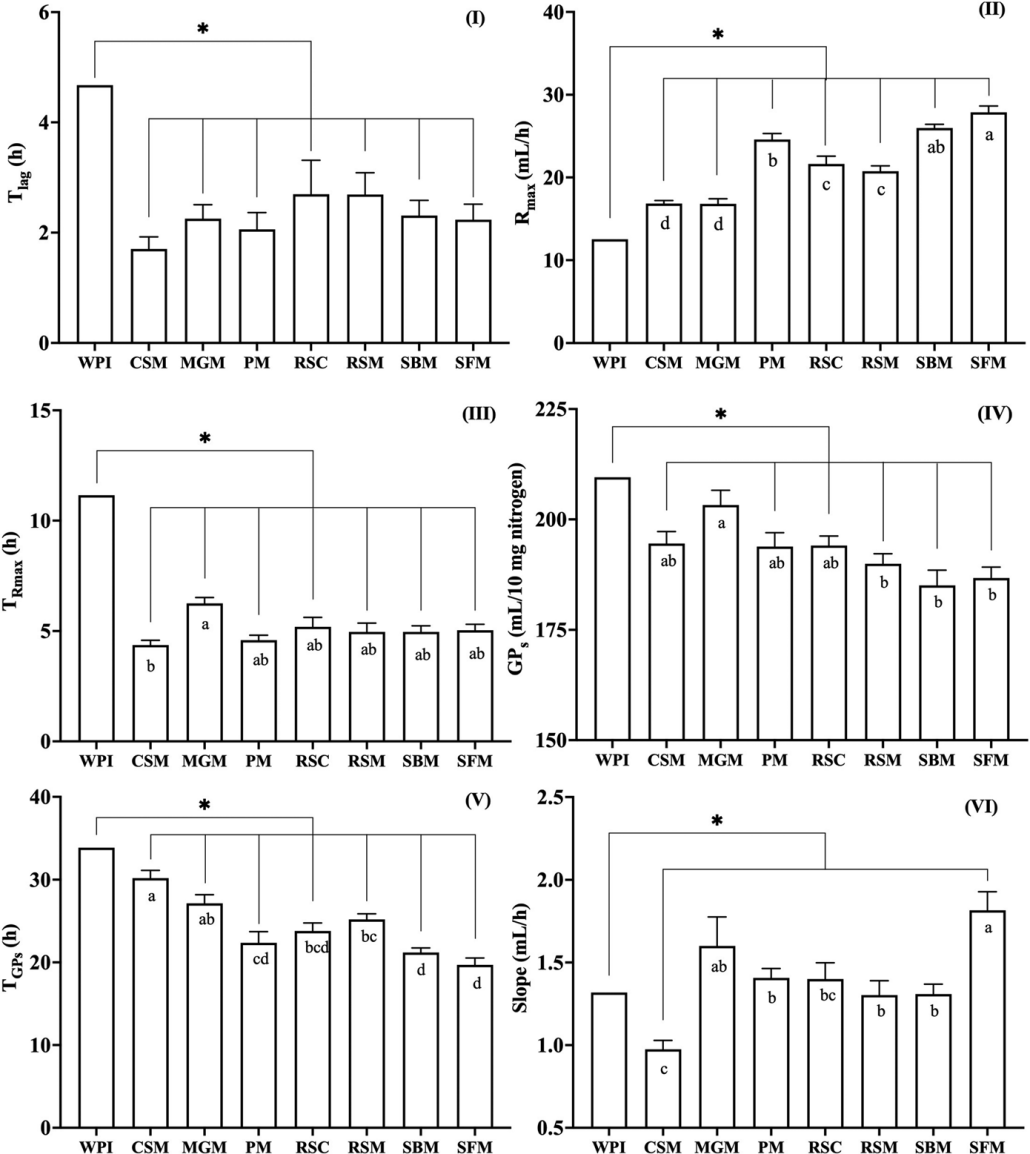
In vitro fermentation parameters of WPI and ileal digesta samples containing 10 mg nitrogen. Means ± SEM of lag time (*T*_lag_, I), maximum GP rate (*R*_max_, II), time when maximum rate occurred (T_Rmax_, III), cumulative GP of protein substrate determined by the model (GP_s_, IV), time when GP_s_ occurred (T_GPs_, V) and slope of the linear line of the model (Slope, VI) during 4 incubation runs. Ileal digesta were obtained from pigs in studies investigating the digestibility of cottonseed meal (CSM), MGM, PM, rapeseed cake (RSC), rapeseed meal (RSM), soybean meal (SBM) and sunflower meal (SFM). Bars with an asterisk showed significant differences between WPI and ileal digesta samples (*P* < 0.05). Bars with different letters within panel showed significant differences between least square means of ileal digesta from pigs fed different protein sources (*P* < 0.05).

Measured cumulative GP curves of the blank, ileal digesta samples and WPI over 48 h is shown in Fig. 1. In general, GP of ileal digesta samples started after approximately 2 h, earlier than WPI (4.7 ± 2.0 h). The difference in *T*_lag_ reached significance for the comparison of WPI with ileal digesta from 4 out of the 7 sources (Fig. 2 panel I). Except for CSM and MGM, all the ileal digesta showed higher *R*_max_ values compared to WPI (12.5 ± 1.0 mL/h, panel II). Highest *R*_max_ value was observed in ileal digesta originating from the ingestion of SFM (27.9 ± 0.8 mL/h) and SBM (26.0 ± 0.5 mL/h). Ileal digesta from pigs fed MGM and CSM fermented much slower (16.8 ± 0.6 and 16.8 ± 0.4 mL/h, respectively) and had a similar rate to WPI. Moreover, T_Rmax_ for all the ileal digesta samples (average 5.1 ± 0.2 h) was shorter compared to WPI (11.2 ± 3.9 h, panel III).

Total GP due to endogenous and dietary undigested protein (GP_s_) in ileal digesta samples (average 192 ± 1.2 mL/10 mg N) as derived from the model were not different to WPI (210 ± 10.3, Fig. 2 panel IV) despite of SBM. Among ileal digesta, samples from pigs fed MGM produced the highest amount of gas (203 ± 3.3 mL/10 mg N), whereas samples from pigs fed SBM produced the lowest amount (185 ± 3.4 mL/10 mg N). Larger differences were detected for T_GPs_ (panel V) values. Significant differences were found in the subsequent microbiota turnover rate (slope, Panel VI), which ranged from 0.99 ± 0.05 mL/h in samples from pigs fed CSM to 1.83 ± 0.1 mL/h for pigs fed SFM.

Fermentation parameters of batches within a protein source are shown in Figs. S1 to S6. No significant differences were detected for *T*_lag_ and GP_s_ between batches. Samples from pigs fed RSM, SFM, PM and RSC showed batch differences in *R*_max_, T_Rmax_, T_GPs_ or slope.

### Nitrogen solubility and molecular size of ileal digesta

**Figure 3.**
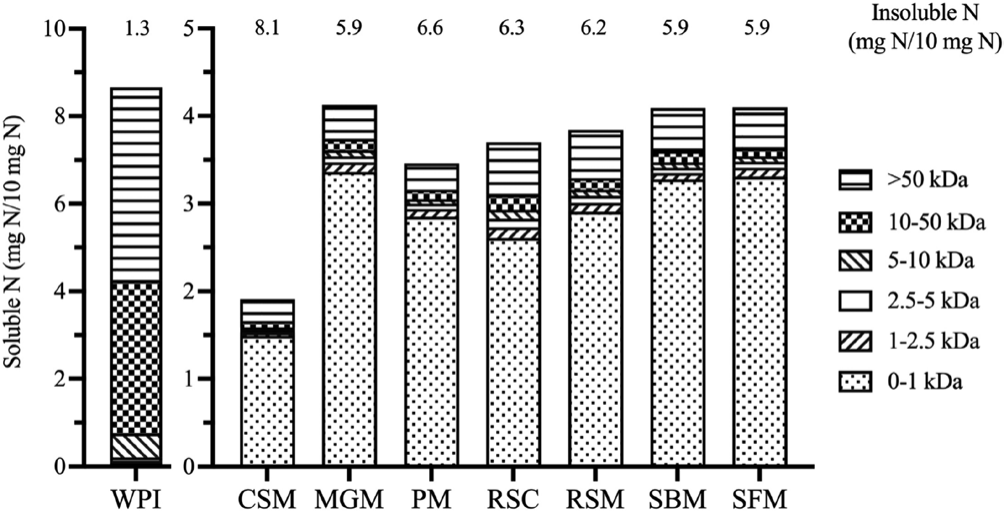
Insoluble nitrogen (N) and molecular mass (kDa) distribution of soluble N in buffer of WPI and ileal digesta samples (*n* = 3, selected batches for one protein source were chosen based on the average values of maximum GP rate). Ileal digesta were obtained from pigs in studies investigating the digestibility of cottonseed meal (CSM), MGM, PM, rapeseed cake (RSC), rapeseed meal (RSM), soybean meal (SBM), and sunflower meal (SFM).

Fig. 3 shows the N solubility and the molecular mass distribution of soluble N of the ileal digesta samples from various protein sources and WPI. In comparison to WPI, the ileal digesta samples displayed a higher proportion of insoluble nitrogenous molecules. Among the ileal digesta samples, those from pigs fed CSM exhibited the lowest levels of soluble proteins (1.9 mg/10 mg N). Within the soluble fraction, peptides sized between 0 and 1 kDa accounted for over half of the content. The stepwise linear regression results between the N solubility and mass distribution of porcine ileal digesta, and their respective in vitro fermentation parameters are shown in Table 2. Both *T*_lag_ and T_Rmax_ exhibited positive associations with the ratio of 10 to 50 kDa N-containing molecules. An increase in the proportion of 0 to 1 kDa N-containing molecules was found to significantly decrease the T_GPs_, while also showing a tendency to increase the *R*_max_. Additionally, the slope (turnover rate) was found negatively associated with the insoluble N level. All the regression equations had a relatively low R^2^.

**Table 2.** Results of stepwise linear regression between in vitro fermentation parameters of 21 selected porcine ileal digesta samples from 7 protein sources and their molecular mass distribution (kDa). Amount of insoluble nitrogen (N) and soluble N with different molecular size range in the total N (mg/10 mg N) were used as initial parameters for selection

Parameter−	Intercept	Selected effect	Slope	*P*-value	*R* ^2^
*T*_lag_ (h)	0.3	10 to 50 kDa	16.6	< 0.01	0.39
*R*_max_ (mL/h)	14.1	0 to 1 kDa	2.8	0.06	0.19
T_Rmax_ (h)	3.4	10 to 50 kDa	30.7	< 0.01	0.36
GP_s_ (mL/10 mg N)	193	NA	NA	NA	NA
T_GPs_ (h)	34.7	0 to 1 kDa	−3.5	< 0.01	0.39
Slope (mL/h)	2.4	Insoluble N	−0.18	0.01	0.31

NA: none of the input variables were determined as a predictor.

^1^ *T*_lag_: the lag time of the start of fermentation (time at which the GP of the substrate bottle surpassed the GP of the blank within a run); *R*_max_: the maximum GP rate by dividing the gas released between 2 consecutive recorded time points by the time interval; T_Rmax_: the time when the *R*_max_ occurred; GP_s_: the GP of the protein source as determined by the modified model; T_GPs_: the time when GP_s_ occurred; slope: the slope of the linear line of the modified model that was fitted to the cumulative GP after T_GPs_.

### Potential gas production of corresponding dietary protein sources

The standardized hindgut GP potential of dietary protein sources (SGP_pro_) varied from 0.46 L/g ingested CP for SBM to 1.18 L/g for MGM (Table 3). When compared to SBM, the relative potential for different sources ranged from 118% for PM to 257% for MGM.

**Table 3.** Potential GP of dietary protein sources in the porcine hindgut. Apparent GP potential (AGP_pro_) was calculated by the in vitro GP from substrate protein as determined by a modified model (GP_s_) of their corresponding ileal digesta samples and apparent ileal digestibility (AID). The standardized GP potential (SGP_pro_) was corrected by GP_s_ of endogenous proteins^1^ and SID. Values were further converted to a relative percentage to soybean meal (SGP_pro:SBM_). All values were averaged from different batches per protein source.

Protein source	GP_s_	AID	SID	AGP_pro_	SGP_pro_	SGP_pro:SBM_
	mL/10 mg nitrogen	%	%	L/g ingested crude protein	L/g ingested crude protein	%
Soybean meal	185	79.9	84.0	0.59	0.46	100
Peanut meal	194	77.0	82.4	0.71	0.54	118
Cottonseed meal	195	75.5	80.3	0.76	0.61	134
Rapeseed cake	194	68.4	77.4	0.98	0.69	150
Sunflower meal	187	66.9	72.5	0.99	0.82	181
Rapeseed meal	190	61.8	71.7	1.16	0.83	179
Maize germ meal	203	37.5	61.6	2.04	1.18	257

^1^GP_s_ of endogenous proteins used for calculation was obtained from previous study (Zhang et al. 2024), except for peanut meal and rapeseed meal where the average value of the other 5 protein sources was used.

## Discussion

The current study included ileal digesta of 58 different ingredients spanning 7 protein sources and provides an initial step in ranking dietary protein sources for their fermentation potential in the large intestine of pigs. As protein is the dominant part of the N in ileal digesta (Table 1), except for the *T*_lag_ where free AA and small peptides may be important, the rate of protein hydrolysis largely determines GP. A N-free but C-rich buffer was used, making N the sole limiting factor. Consequently, differences in in vitro GP curves should be attributed solely to N accessibility whereby N utilization by the microbiota depends on the type of N-containing molecules, their availability for absorption, and their role in metabolism.

Ileal digesta samples behaved differently during fermentation, especially in terms of *R*_max_, T_GPs_ and GP_s_ when the same amount of N was provided. Ileal digesta of pigs fed 5 out of the 7 protein sources had a greater *R*_max_ than intact WPI (Fig. 2. Panel II). These findings parallel our previous study (Zhang et al. 2024) that showed that ileal digesta from pigs fed N-free diets had a high *R*_max_ compared to intact WPI in an environment with excess carbohydrates. Another study showed that ileal digesta from pigs fed WPI showed the highest *R*_max_ of 11 protein sources (Lammers-Jannink et al., 2023) which can be explained by the relative large endogenous N fraction in ileal digesta which is known to have a high rate of fermentation (Zhang et al. 2024) as WPI is almost 100% digestible (Mathai et al., 2017). It also found that an increased degree of hydrolysis of WPI led to a greater *R*_max_ (Zhang et al. 2024). This effect is attributed to the increased ratio of peptides present in hydrolyzed WPI, requiring less hydrolysis to form absorbable AA and di- or tripeptides. Consequently, the rate of hydrolysis, representing the speed at which AA become available for metabolism, plays a pivotal role in determining the GP profile.

After absorption, AA can be incorporated into microbial protein or serve as a substrate for catabolic pathways where they can be deaminated with subsequent production of ammonia and keto-acids. The latter acids can proceed through decarboxylation reactions to generate short-chain fatty acids (**SCFA**) including butyrate, acetate, propionate, lactate, succinate, and formate (Diether and Willing, 2019). Quantitatively, SCFA and ammonia are the major products of AA fermentation (Smith and Macfarlane, 1998). In the in vitro system used here, the SCFA generated produce CO_2_ when buffered, thereby increasing GP. While the in vitro system may not directly provide information on the subsequent rate of AA deamination or decarboxylation reactions, where AA are utilized as an energy source, it nonetheless elucidates the crucial hydrolysis step essential for subsequent AA metabolism during microbial growth and fermentation. As protein hydrolysis has been shown to be the rate-limiting step in protein fermentation (Breure and van Andel, 1984; Flotats et al., 2006), this system offers insights into the rate of hydrolysis of peptide bonds from the available protein in the substrate.

The hydrolysis rate can vary among different AA. Aspartate, Ser, Arg and Thr were reported to be the most rapidly metabolized AA by human microbiota (Smith and Macfarlane, 1998). Furthermore, AA are metabolized by microbiota depending on protein composition. Metabolism can range from 20% for Asp and Tyr in gelatin to 100% for Arg and Asp in casein (Bevilacqua et al., 2020). Lower degradation of certain AA like Tyr was considered a strategy by bacteria to avoid further accumulation of aromatic fatty acids, which are more toxic than the aliphatic ones (Kim et al., 2001). Apart from hydrolysis rate, the potential of microbiota in using different AA also varies. For instance, there is a lower diversity of bacteria in the hindgut of pigs utilizing Tyr compared with Trp and Phe, while a large number of bacteria participated in Phe synthesis (Ma, He, and Zhu, 2016). Therefore, the abundance of certain AA in protein sources that can serve as substrates for specific microbial groups, influences the rate and extent of fermentation. Although, the variation in AA profiles of the undigested dietary and endogenous proteins present in ileal digesta can be expected to contribute to differences in the current study, a clear relation between the AA profile and GP parameters was not found.

Due to energetical efficiency (Wright, 1967), intestinal microbiota may display a preference for peptides over AA, as indicated by higher rates of metabolite production (Smith and Macfarlane, 1998). For this reason, we investigated the relation between molecular size and solubility with fermentation parameters. We observed a negative relation between small peptides (< 1 kDa) and T_GPs_ and a trend for a positive relation between with *R*_max_, whereas 10 to 50 kDa fraction delayed *T*_lag_ and T_Rmax_. This finding supports our hypothesis that smaller proteins have a higher degradation rate and thereby can achieve their T_GPs_ earlier. These results, however, differs from our previous study (Zhang et al. 2024) where the insoluble endogenous protein in ileal digesta was positively related with *R*_max_. One underlying mechanism is the distinct composition of undigested dietary protein compared to endogenous protein, possibly leading to differences in accessibility of microbial enzymes. It was reported that the specificity and efficiency of enzymatic hydrolysis depend on the type of microbial protease, as different proteases have distinct substrate preferences and cleavage specificities (Rao et al., 1998). Like trypsin preferentially cleaves peptide bonds on the carboxyl side of Lys and Arg residues (Olsen, Ong, and Mann, 2004), proteases originated from different microorganisms have been shown to rapidly hydrolyze peptide bonds containing amino groups like Leu and Phe (Morihara, Tsuzuki, and Oka, 1968). The enzymes can also encounter structural challenges with other peptides such as those containing sulphur bonds (Darby and Creighton, 1995). Hence, it is possible that in our previous study, certain unidentified mechanisms enabled the fecal microbiota to utilize endogenous proteins from the same species. However, within the digesta mixture, the molecular size of soluble proteins may still be a significant factor influencing the GP patterns.

When comparing the ileal digesta samples, we observed that *R*_max_ was approximately 1.7 times as high for SFM compared to CSM whereas GP_s_ only varied 1.1-fold between the highest (MGM) and lowest (SBM) sample. The variation in cumulative GP between samples was considerably lower to the previous findings (Cone et al., 2005; Lammers-Jannink et al., 2023) who also investigated in vitro GP from protein with easily fermentable carbohydrates (5.4 g/L). In their studies, maximal differences of 1.4-fold in asymptotic GP and 1.8-fold for cumulative GP at 20 h were observed. The relative lower variation for GP_s_ observed in the current study is probably attributed to differences in methodology. The previously mentioned studies either compared GP at the same time point (20 h) for all the samples or used all the 48-h data including potentially microbiota turnover to estimate asymptotic value. However, the full fermentation potential is represented by GP_s_ which occurs at significantly different time points (Fig 2. Panel V) and before 48 h as proteins are relatively rapidly hydrolyzed and subsequently metabolized into SCFA. In the previous study (Cone et al., 2005), the GP per mg of N was lower compared to the current study (10 to 18 vs. 19 to 21 mL/mg N). This may be related to difference in the buffer carbohydrate content (5.4 vs 21.6 g/L), or the inoculum used. Nevertheless, we find similar variation range in *R*_max_ with both studies.

Differences in protein fermentation between protein sources have also been shown in vivo. A recent study (Noorman et al., 2023) demonstrated that variations in dietary protein sources, along with sanitary status, influenced the colonic flow of protein fermentation metabolites in piglets. Similarly, a study involving pigs fed 18 different foods having a low to high protein digestibility (containing 100 g protein/kg dry matter) found varying concentrations of tryptophan metabolites (*P* < 0.05) in the feces (Huang et al., 2023). Likewise, piglets fed concentrated degossypolized cottonseed protein exhibited elevated concentrations of valeric acid (*P* = 0.05) and branched-chain fatty acids (*P* = 0.06) in the colon compared to other studied diets like fish meal (Li et al., 2019). Comparing these in vivo findings to the current study is challenging as most in vivo studies rely on single-point measurements, lacking dynamic profiles such as the GP rate of protein fermentation. Nevertheless, the in vivo studies confirm significant variations in fermentation potential between protein sources in the animal gut, underscoring the importance of evaluating these differences for effective diet management. It also highlights the potential of in vitro GP systems for providing a more comprehensive, time-sensitive analysis of protein fermentation dynamics.

Our study aimed to rank protein sources based on their fermentation potential by measuring the fermentation of undigested dietary protein and specific endogenous protein losses. To achieve this, we introduced a novel metric, SGP_pro_, which quantifies the fermentation potential per gram of CP ingested, corrected for the GP due to basal endogenous protein losses. This new metric allows for a more accurate comparison of fermentation potential across different protein sources by accounting for the inherent variation in endogenous protein contributions. Among the dietary protein sources, SGP_pro_ ranged from 0.46 for SBM to 1.18 L/g ingested CP for MGM (Table 3), assuming a linear relationship between maximum GP and available protein level. The low fermentation potential of SBM is achieved through undigested protein fraction being challenging for microbiota to utilize in combination with its high digestibility. As in our calculation of SGP_pro_, GP_s_ is used and there are minor differences between dietary protein sources in term of GP_s_, the substantial variation observed results predominantly from the difference in N digestibility between protein sources. More substantial differences were observed in T_GPs_ rather than GP_s_. Protein sources that have similar GP_s_ can have different rates of fermentation and can be, therefore, expected to behave differently in the gut due to variation in timing (T_GPs_) and *R*_max_. All the substrates in the current study seems to be fermented completely between 20 to 30 h under the experimental conditions employed. The mean retention time in the large intestine of growing pigs was found to be between 26 to 40 h and 36 to 44 h for liquid and solid particles, respectively, dependent on the dietary fiber content (Wilfart et al., 2007). It is reported that in the large intestine of pigs (63 ± 3 kg), the shortest apparent mean retention time was observed in the cecum (2 h), with values for the proximate, mid and distal colon being 5.5, 4.8 and 3.1 h, respectively (Low et al., 2020). Our data may provide insights into which segments of the large intestine significant protein fermentation can occur.

The current study was designed to maximize N utilization in a C-rich environment. One strength of our study is that a standardized method was used to compare the most important protein sources used in pig nutrition and that multiple batches per protein source were investigated, which is much more challenging in vivo. The use of ileal digesta, the material actually flowing into the cecum/colon under practical feeding conditions, enhances the credibility of our findings to an in vivo scenario. However, intestinal absorption is not simulated in the in vitro system and the assumption of linearity between GP and N levels cannot be assumed under in vivo conditions. Also, the assumption of pure microbial turnover at the end has not been verified. Another advantage is that multiple batches of a protein source were investigated. It is well-known that variations in sourcing and processing methods can introduce variability in the nutritional characteristics of protein sources and their digestibility (Rojas and Stein, 2017). Obtaining average data across a broad range of batches allows for a representative estimation of the protein source. Furthermore, our consideration of relative effects compared to SBM facilitates easier comparisons in translating findings to in vivo settings. As recommended by the Food and Agriculture Organization of the United Nations (FAO, 2011), standardized ileal AA digestibility values of growing pigs can be used to determine the digestible indispensable AA score (DIAAS) of protein sources for human consumption. As such, the current study, in addition to its direct applicability to pigs, also advances our understanding of protein fermentation potential from different sources for humans.

Sustainability and precision in pig feed formulation have gained increasing attention (Stein et al., 2016). Many pig diets rely on cereal grains and soy byproducts from South America, raising concerns about sustainability and competition with human food resources. The Netherlands has adopted circular agriculture (LNV, 2018) to enhance sustainability, aligning with the shift toward alternative protein sources in human nutrition (Biesbroek et al., 2023). Protein digestibility varies significantly between sources, meaning diets with equivalent digestible protein levels may differ in undigested protein supply to the large intestine, influencing fermentation and metabolite production in both pigs and humans. Our study provides a valuable database on protein fermentation potential for the first time to guide future research and further innovations in feed formulation. The new SGP_pro_ metric can be integrated into feed and dietary strategies, considering factors like age, health status, and nutritional goals. Tailoring fermentation potential could help optimize gut health in piglets, pigs with digestive issues, as well as humans with conditions like irritable bowel syndrome or inflammatory bowel disease (Gilbert et al., 2018). The current discussion for applications is based on in vitro assessments of fermentability, while in vivo interactions with carbohydrates, which are oversupplied in the current in vitro GP system, can alter protein fermentation. Furthermore, here the focus was only on protein fermentation in the large intestine, but there may be already some protein fermentation occurring in the stomach and small intestine as reviewed by Zhang et al. (2020). Additionally, microbiota composition is influenced by numerous factors, including animal and environmental conditions, highlighting the importance of considering individual differences. Variations within protein sources were observed in GP patterns, suggesting the need for ingredient-specific fermentability testing. Lastly, while understanding protein fermentability is important, the threshold for harmful fermentation and the impact of specific metabolites remain unclear and context dependent. The relationship between GP kinetics and metabolite production requires further exploration.

## Conclusion

Significant differences in the rate of in vitro protein fermentation were observed, although total fermentation was comparable across ileal digesta from pigs fed different protein sources. Notably, porcine ileal digesta exhibited greater in vitro protein fermentability than intact whey protein isolate. Nitrogen solubility and molecular mass distribution were key factors influencing the in vitro fermentation patterns of ileal digesta. The model developed in this study, using the novel metric standardized gas production potential (**SGP**_**pro**_), offers a method for predicting protein fermentation potential and its potential health effects when comparing different protein sources in pig nutrition. This approach provides valuable insights into optimizing dietary protein selection for improved gut health and fermentation outcomes.

## Supplementary Material

skaf119_suppl_Supplementary_Material

## Abbreviations

AA: amino acid
Ai: the asymptotic gas production
AGPpro: the apparent hindgut gas production potential of a dietary protein source
AID: apparent ileal digestibility
Bi: the time after incubation at which half of the asymptotic amount of gas has been formed
Ci: a constant determining the sharpness of the switching characteristic of the profile
CP: crude protein
CSM: cottonseed meal
GP: gas production
GPc: the amount of gas produced per 10 mg N of sample incubated during time
GPendo: the gas production potential of the corresponding endogenous proteins
GPs: the total gas production from the protein source
kDa: kilodalton
MGM: maize germ meal
N: nitrogen
PM: peanut meal
*R*_max_: maximum gas production rate
RSC: rapeseed cake
RSM: rapeseed meal
SBM: soybean meal
SCFA: short-chain fatty acids
SEC: size exclusion chromatography
SFM: sunflower meal
SGPpro: the standardized gas production potential of a dietary protein source
SID: standardized ileal digestibility
TGPs: the time when GPs occurred
Tlag: the lag time of the start of fermentation
*T*_*R*max_: the time when *R*_max_ occurred
WPI: whey protein isolate
WPIH: whey protein isolate hydrolysate
