# Supplementary material for: In vitro fermentation potential of undigested dietary protein
Source: J Anim Sci. 2025 Apr 17;103:skaf119. doi: 10.1093/jas/skaf119 (PMC12080548; doi:10.1093/jas/skaf119)
Supplement: skaf119_suppl_Supplementary_Material [file skaf119_suppl_supplementary_material.docx]

***In Vitro* Fermentation Potential of Undigested Dietary Protein**

Hanlu Zhang^a,b^, John W. Cone^a^, Arie K. Kies^c^, Wouter H. Hendriks^a^ and Nikkie van der Wielen^a,d,*^

^a^ Animal Nutrition Group, Department of Animal Sciences, Wageningen University & Research, Wageningen, The Netherlands;

^b^ State Key Laboratory of Animal Nutrition, College of Animal Science and Technology, China Agricultural University, Beijing, China;

^c^ ArieKiesAdvies, Druten, The Netherlands;

^d^ Division of Human Nutrition and Health, Department of Agrotechnology and Food Sciences, Wageningen University & Research, Wageningen, The Netherlands.

**^*^**Correspondence: [nikkie.vanderwielen@wur.nl](mailto:nikkie.vanderwielen@wur.nl)

**Running title:** Ileal Digesta Fermentation in Growing Pigs

**Keywords:** Protein fermentation; plant protein; pig; gas production system; curve fitting

| Supplemental Table 1. Mean (min, max) nutrient composition (%, dry matter basis) of protein ingredients originating from different sources with different batches and their ileal digestibility in growing pigs. | | | | | | | |
| --- | --- | --- | --- | --- | --- | --- | --- |
| Source  Item^*^ | Cottonseed meal | Maize germ meal | Peanut meal | Rapeseed cake | Rapeseed meal | Soybean meal | Sunflower meal |
| Crude protein | 50.2 (39.1, 56.9) | 20.2 (18.4, 24.4) | 55.2 (51.7, 59.2) | 40.9 (36.8, 44.7) | 42.3 (40.3, 43.6) | 50.2 (45.3, 53.7) | 33.7 (29.3, 39.1) |
| Ether extract | 0.7 (0.4, 1.1) | 1.6 (0.4, 2.8) | 0.9 (0.3, 1.7) | 10.8 (9.5, 12.6) | 1.4 (0.7, 1.8) | 0.9 (0.2, 1.4) | 3.3 (0.9, 11.3) |
| NDF | 36.6 (29.4, 46.3) | 50.9 (40.7, 55.4) | 22 (14.8, 28.2) | 38 (31.0, 47.1) | 36.2 (29.9, 41.0) | 15.3 (12.1, 19.5) | 44.6 (38.2, 55.4) |
| ADF | 15.3 (9.6, 22.9) | 14.8 (10.9, 18.9) | 7.8 (6.7, 9.6) | 21.9 (16.7, 27.2) | 23.9 (21.1, 28.2) | 7.2 (4.1, 11.9) | 28.6 (24.6, 37.3) |
| Ash | 6.1 (5.4, 6.8) | 3.2 (1.6, 5.4) | 6.9 (6.3, 8.1) | 7.7 (6.8, 8.3) | 8.3 (7.7, 8.9) | 6.6 (6.2, 6.9) | 6.9 (5.5, 8.3) |
| Calcium | 0.2 (0.2, 0.3) | 0.1 (0, 0.2) | 0.3 (0.1, 0.4) | 0.8 (0.7, 0.9) | 0.9 (0.8, 1.1) | 0.4 (0.2, 0.8) | 0.3 (0.2, 0.5) |
| Phosphorus | 1.0 (0.7, 1.1) | 0.4 (0.3, 0.8) | 0.8 (0.7, 0.9) | 1.3 (1.2, 1.3) | 1.0 (0.9, 1.3) | 0.8 (0.6, 0.9) | 0.9 (0.6, 1.2) |
| GE (MJ/kg) | 17.4 (16.9, 18.2) | 19.2 (18.6, 19.7) | 17.1 (16.7, 17.4) | 21.2 (20.9, 21.9) | 19.2 (19, 19.4) | 19.5 (19.2, 20.0) | 19.5 (18.7, 21.4) |
| AID_CP_ (%) | 75.5 (72.0, 78.5) | 37.5 (23.9, 54.9) | 77 (70.2, 82.0) | 68.4 (60.0, 73.0) | 61.8 (57.3, 66.2) | 79.9 (77.3, 82.3) | 66.9 (59.7, 74.7) |
| SID_CP_ (%) | 80.3 (76.7, 82.9) | 61.6 (47.9, 80.6) | 82.4 (75.7, 87.7) | 77.4 (69.6, 81.7) | 71.7 (66.9, 76.4) | 84.1 (82.1, 87.3) | 72.5 (66.3, 79.3) |
| ^*^**NDF: neutral detergent fibre; ADF: acid detergent fibre; GE: gross energy; AID_CP_: apparent ileal digestibility of crude protein; SID_CP_: standardised ileal digestibility of crude protein.** | | | | | | | |

| Supplemental Table 2. Mean (min, max) amino acid content^*^ (mg/10 mg N) of pooled ileal digesta of pigs fed seven protein sources. | | | | | | | |  |
| --- | --- | --- | --- | --- | --- | --- | --- | --- |
| Source  Amino acid | Cottonseed meal | Maize germ meal | Peanut meal | Rapeseed cake | Rapeseed meal | Soybean meal | Sunflower meal | |
| Alanine | 0.71 (0.65, 0.79) | 0.51 (0.43, 0.60) | 0.52 (0.47, 0.55) | 0.53 (0.41, 0.60) | 0.48 (0.47, 0.50) | 0.65 (0.59, 0.72) | 0.59 (0.52, 0.75) | |
| Arginine | 1.14 (1.07, 1.24) | 0.41 (0.35, 0.52) | 0.70 (0.64, 0.75) | 0.72 (0.60, 0.79) | 0.69 (0.64, 0.75) | 0.69 (0.63, 0.75) | 0.76 (0.56, 0.84) | |
| Aspartic acid | 1.09 (1.03, 1.23) | 0.63 (0.58, 0.68) | 1.08 (0.95, 1.23) | 0.78 (0.68, 0.85) | 0.80 (0.76, 0.85) | 1.11 (1.01, 1.24) | 0.98 (0.84, 1.38) | |
| Cysteine | 0.17 (0.17, 0.19) | 0.19 (0.17, 0.28) | 0.13 (0.11, 0.16) | 0.17 (0.13, 0.20) | 0.19 (0.17, 0.20) | 0.19 (0.17, 0.21) | 0.16 (0.14, 0.23) | |
| Glutamic acid | 1.30 (1.23, 1.45) | 0.87 (0.82, 0.93) | 1.33 (1.19, 1.42) | 0.91 (0.81, 0.96) | 1.00 (0.96, 1.06) | 1.56 (1.24, 1.87) | 1.28 (1.10, 1.73) | |
| Glycine | 0.96 (0.87, 1.08) | 1.00 (0.86, 1.29) | 1.23 (1.10, 1.31) | 0.97 (0.74, 1.05) | 0.89 (0.84, 0.94) | 0.82 (0.75, 0.92) | 1.26 (1.05, 1.57) | |
| Histidine | 0.47 (0.44, 0.53) | 0.27 (0.25, 0.32) | 0.34 (0.32, 0.36) | 0.38 (0.34, 0.43) | 0.31 (0.28, 0.41) | 0.34 (0.30, 0.38) | 0.40 (0.35, 0.59) | |
| Isoleucine | 0.31 (0.28, 0.35) | 0.17 (0.15, 0.18) | 0.22 (0.20, 0.26) | 0.24 (0.19, 0.29) | 0.25 (0.23, 0.27) | 0.27 (0.25, 0.31) | 0.27 (0.24, 0.32) | |
| Leucine | 0.54 (0.49, 0.62) | 0.30 (0.27, 0.35) | 0.37 (0.33, 0.40) | 0.44 (0.36, 0.52) | 0.39 (0.36, 0.42) | 0.48 (0.44, 0.54) | 0.45 (0.40, 0.57) | |
| Lysine | 0.92 (0.82, 1.03) | 0.34 (0.32, 0.38) | 0.52 (0.50, 0.55) | 0.64 (0.56, 0.72) | 0.68 (0.63, 0.73) | 0.63 (0.56, 0.71) | 0.50 (0.42, 0.70) | |
| Methionine | 0.10 (0.09, 0.12) | 0.05 (0.03, 0.10) | 0.04 (0.03, 0.04) | 0.07 (0.07, 0.08) | 0.07 (0.06, 0.07) | 0.05 (0.05, 0.06) | 0.08 (0.06, 0.11) | |
| Phenylalanine | 0.25 (0.23, 0.28) | 0.12 (0.11, 0.13) | 0.17 (0.16, 0.19) | 0.18 (0.15, 0.19) | 0.17 (0.15, 0.18) | 0.25 (0.23, 0.27) | 0.19 (0.17, 0.25) | |
| Proline | 0.63 (0.44, 0.82) | 1.12 (0.69, 1.56) | 0.35 (0.28, 0.48) | 0.74 (0.57, 1.05) | 0.79 (0.64, 0.94) | 0.37 (0.34, 0.40) | 0.34 (0.24, 0.65) | |
| Serine | 0.48 (0.46, 0.55) | 0.34 (0.30, 0.46) | 0.43 (0.39, 0.45) | 0.44 (0.35, 0.51) | 0.42 (0.40, 0.44) | 0.43 (0.39, 0.46) | 0.46 (0.40, 0.66) | |
| Threonine | 0.45 (0.41, 0.50) | 0.34 (0.29, 0.47) | 0.30 (0.28, 0.32) | 0.40 (0.33, 0.45) | 0.40 (0.39, 0.42) | 0.39 (0.36, 0.43) | 0.36 (0.33, 0.47) | |
| Tryptophan | 0.12 (0.11, 0.14) | 0.07 (0.06, 0.09) | 0.10 (0.09, 0.12) | 0.11 (0.10, 0.14) | 0.10 (0.09, 0.13) | 0.12 (0.11, 0.13) | 0.10 (0.09, 0.13) | |
| Tyrosine | 0.14 (0.11, 0.15) | 0.08 (0.07, 0.09) | 0.12 (0.10, 0.14) | 0.13 (0.10, 0.14) | 0.14 (0.12, 0.17) | 0.09 (0.08, 0.11) | 0.08 (0.06, 0.10) | |
| Valine | 0.39 (0.33, 0.42) | 0.32 (0.28, 0.37) | 0.40 (0.36, 0.42) | 0.38 (0.30, 0.44) | 0.40 (0.36, 0.43) | 0.37 (0.33, 0.43) | 0.46 (0.42, 0.60) | |
| ^*^Values used for calculation were obtained from previous studies (Ma et al. 2019; Li et al. 2015a; Liu et al. 2015; Li et al. 2014; Li et al. 2015b; 2017; Zhang et al. 2019). | | | | | | | |  |

Supplemental Fig. 1. Mean ± SEM lag time (T_lag_) of *in vitro* gas production of ileal digesta (n=4) containing 10 mg nitrogen from pigs fed seven different protein sources. Ileal digesta originated from pigs in studies investigating the digestibility of different batches of cottonseed meal (CSM, n=10), maize germ meal (MGM, n=8), rapeseed cake (RSC, n=4), peanut meal (PM, n=7), rapeseed meal (RSM, n=9), soybean meal (SBM, n=11) and sunflower meal (SFM, n=9).

Supplemental Fig. 2. Mean ± SEM maximum gas production rate (R_max_) determined from the *in vitro* gas production of ileal digesta (n=4) containing 10 mg nitrogen from pigs fed seven different protein sources. Ileal digesta originated from studies investigating the digestibility of different batches of cottonseed meal (CSM, n=10), maize germ meal (MGM, n=8), rapeseed cake (RSC, n=4), peanut meal (PM, n=7), rapeseed meal (RSM, n=9), soybean meal (SBM, n=11) and sunflower meal (SFM, n=9). Bars within protein source with different letters differ (*P* < 0.05).

Supplemental Fig. 3. Mean ± SEM time when maximum fermentation rate occurred (T_Rmax_) determined from the *in vitro* gas production of ileal digesta (n=4) containing 10 mg nitrogen from pigs fed seven different protein sources. Ileal digesta originated from studies investigating the digestibility of different batches of cottonseed meal (CSM, n=10), maize germ meal (MGM, n=8), rapeseed cake (RSC, n=4), peanut meal (PM, n=7), rapeseed meal (RSM, n=9), soybean meal (SBM, n=11) and sunflower meal (SFM, n=9). Bars within protein source with different letters differ (*P* < 0.05).

Supplemental Fig. 4. Mean ± SEM cumulative gas production (GP_s_) as determined by the model fitted to the *in vitro* gas production of ileal digesta (n=4) containing 10 mg nitrogen from pigs fed seven different protein sources. Ileal digesta originated from studies investigating the digestibility of different batches of cottonseed meal (CSM, n=10), maize germ meal (MGM, n=8), rapeseed cake (RSC, n=4), peanut meal (PM, n=7), rapeseed meal (RSM, n=9), soybean meal (SBM, n=11) and sunflower meal (SFM, n=9).

Supplemental Fig. 5. Mean ± SEM time when cumulative gas production occurred from the model fitted to the *in vitro* gas production of ileal digesta (n=4) containing 10 mg nitrogen from pigs fed seven different protein sources. Ileal digesta originated from studies investigating the digestibility of different batches of cottonseed meal (CSM, n=10), maize germ meal (MGM, n=8), rapeseed cake (RSC, n=4), peanut meal (PM, n=7), rapeseed meal (RSM, n=9), soybean meal (SBM, n=11) and sunflower meal (SFM, n=9). Bars within protein source with different letters differ (*P* < 0.05).

Supplemental Fig. 6. Mean ± SEM regression coefficients of the linear line from the model fitted to the *in vitro* gas production of ileal digesta (n=4) containing 10 mg nitrogen from pigs fed seven different protein sources. Ileal digesta originated from studies investigating the digestibility of different batches of cottonseed meal (CSM, n=10), maize germ meal (MGM, n=8), rapeseed cake (RSC, n=4), peanut meal (PM, n=7), rapeseed meal (RSM, n=9), soybean meal (SBM, n=11) and sunflower meal (SFM, n=9).

Li, P L, Y F Chen, Z Q Lyu, S B Yu, F Wu, B B Huang, L Liu, and C H Lai. 2017. “Concentration of Metabolizable Energy and Digestibility of Amino Acids in Chinese Produced Dehulled Double-Low Rapeseed Expellers and Non-Dehulled Double-Low Rapeseed Co-Products Fed to Growing-Finishing Pigs.” *Animal Feed Science and Technology* 234:10–19. https://doi.org/https://doi.org/10.1016/j.anifeedsci.2017.09.001.

Li, P L, F Wu, Y F Chen, J R Wang, P P Guo, Z C Li, L Liu, and C H Lai. 2015. “Determination of the Energy Content and Amino Acid Digestibility of Double-Low Rapeseed Cakes Fed to Growing Pigs.” *Animal Feed Science and Technology* 210:243–53. https://doi.org/https://doi.org/10.1016/j.anifeedsci.2015.10.012.

Li, Qingyun, Xiangshu Piao, Jundi Liu, Zhikai Zeng, Sai Zhang, and Xinjian Lei. 2014. “Determination and Prediction of the Energy Content and Amino Acid Digestibility of Peanut Meals Fed to Growing Pigs.” *Archives of Animal Nutrition* 68 (3): 196–210. https://doi.org/10.1080/1745039X.2014.910970.

Li, Zhongchao, Xiaoxiao Wang, Panpan Guo, Ling Liu, Xiangshu Piao, Hans H Stein, Defa Li, and Changhua Lai. 2015. “Prediction of Digestible and Metabolisable Energy in Soybean Meals Produced from Soybeans of Different Origins Fed to Growing Pigs.” *Archives of Animal Nutrition* 69 (6): 473–86. https://doi.org/10.1080/1745039X.2015.1095461.

Liu, J D, Q Y Li, Z K Zeng, P Li, X Xu, H L Wang, S Zhang, and X S Piao. 2015. “Determination and Prediction of the Amino Acid Digestibility of Sunflower Seed  Meals in Growing Pigs.” *Asian-Australasian Journal of Animal Sciences* 28 (1): 86–94. https://doi.org/10.5713/ajas.14.0109.

Ma, Xiaokang, Jiangxu Hu, Qinghui Shang, Hansuo Liu, and Xiangshu Piao. 2019. “Chemical Composition, Energy Content and Amino Acid Digestibility in Cottonseed Meals Fed to Growing Pigs.” *Journal of Applied Animal Research* 47 (1): 280–88. https://doi.org/10.1080/09712119.2019.1626241.

Zhang, Zeyu, Zhaoyu Liu, Shuai Zhang, Changhua Lai, Dongli Ma, and Chengfei Huang. 2019. “Effect of Inclusion Level of Corn Germ Meal on the Digestible and Metabolizable Energy and Evaluation of Ileal AA Digestibility of Corn Germ Meal Fed to Growing Pigs1.” *Journal of Animal Science* 97 (2): 768–78. https://doi.org/10.1093/jas/sky469.
